# Isolation of a novel multiple-heavy metal resistant *Lampropedia aestuarii* GYF-1 and investigation of its bioremediation potential

**DOI:** 10.1186/s12866-023-03093-4

**Published:** 2023-11-07

**Authors:** Lan Yu, Tao Zhang, Jiacheng Yang, Rongfei Zhang, Junbo Zhou, Fan Ding, Chaogang Shao, Rongkai Guo

**Affiliations:** 1 College of Life Sciences, Huzhou University, Huzhou, 313000 P.R. China; 2 Shanghai Institute of Plant Physiology and Ecology, Chinese Academy of Sciences, Shanghai, 200032 P.R. China; 3 Shenzhen MSU-BIT University, Shenzhen, 518172 China

**Keywords:** *Lampropedia aesturaii*, Mixed heavy metal stress adaptation, Genome analysis, Relative gene expression, Bioremediation

## Abstract

**Background:**

Heavy metal contamination has been a severe worldwide environmental issue. For industrial pollutions, heavy metals rarely exist as singular entities. Hence, researches have increasingly focused on the detrimental effect of mixed heavy metal pollution. Genome analysis of *Lampropedia* strains predicted a repertoire of heavy metal resistance genes. However, we are still lack of experimental evidence regarding to heavy metal resistance of *Lampropedia*, and their potential in mixed heavy metal removal remain elusive.

**Results:**

In this study, a *Lampropedia aestuarii* strain GYF-1 was isolated from soil samples near steel factory. Heavy metal tolerance assay indicated *L. aestuarii* GYF-1 possessed minimal inhibition values of 2 mM, 10 mM, 6 mM, 4 mM, 6 mM, 0.8 mM, and 4 mM for CdCl_2_, K_2_CrO_4_, CuCl_2_, NiCl_2_, Pb(CH_3_COO)_2_, ZnSO_4_, and FeCl_2_, respectively. The biosorption assay demonstrated its potential in soil remediation from mixed heavy metal pollution. Next the draft genome of *L. aestuarii* GYF-1 was obtained and annotated, which revealed strain GYF-1 are abundant in heavy metal resistance genes. Further evaluations on differential gene expressions suggested adaptive mechanisms including increased lipopolysaccharides level and enhanced biofilm formation.

**Conclusion:**

In this study, we demonstrated a newly isolated *L. aestuarii* GYF-1 exhibited mixed heavy metal resistance, which proven its capability of being a potential candidate strain for industrial biosorption application. Further genome analysis and differential gene expression assay suggest enhanced LPS and biofilm formation contributed to the adaptation of mixed heavy metals.

**Supplementary Information:**

The online version contains supplementary material available at 10.1186/s12866-023-03093-4.

## Introduction

Heavy metal contamination has been a severe worldwide environmental issue due to their toxicity, accumulative, and nonbiodegradable properties. The primary cause of heavy metal pollution has emerged from industrial activities, such as mining, electroplating, paints and pigments, batteries, tanning and textile, steel industries. Use of pesticides, insecticides, fertilizers in agricultural fields have been secondary source of heavy metal contamination [1]. Recently, research focuses on metal pollution have trends to shift from single metals to mixed metals. For industrial pollutions, heavy metals rarely exist as singular entities. Hence, researches have increasingly focused on the detrimental effect of mixed heavy metal pollution to ecosystem and health of living system. For example, heavy metal mixture induces global iron starvation resulted in decreased activity of biological nitrate removal [2]. Exposure to mixed heavy metals is negatively associated with renal function via oxidative stress disorder [3]. Accumulation of mixed heavy metals is highly related to the occurrence of cancer [4]. Thus, effective approaches must be taken for remediation of mixed heavy metals contamination.

Microorganisms have developed various mechanisms for adaptation of mixed heavy metal stress, resulting in an eco-friendly and cost-effective strategy called biosorption. The detoxification of mixed heavy metals includes biosorption via production of extracellular polymeric substances (EPS), efflux of toxic metals by active transporters, intracellular sequestration, surface precipitation, metal reduction. To date, many bacterial groups are considered as potential bioagents for mixed heavy metal removal, such as *Bacillus sp.*, *Pseudomonas sp.*, *Alcaligenes sp.*, *Rhizopus sp.*, *Sphingomonas sp.*, *Azospira sp.* and *Cupriavidus sp.*, etc [5, 6]. Therefore, discovery of novel bacteria contributes to mixed heavy metal removal is of great importance, which would benefit not only to better understanding of the bacterial adaptation mechanisms, but also to development of new biosorption strategies.

*Lampropedia spp*. is a Gram-negative, Neisser-positive, non-spore forming coccus. *L. hyalina* was firstly isolated from polluted water sample by Schroeter in 1886 [7]. Since then, the other three *L. hyalina* strains, ATCC 11,041, ATCC 43,383, DSM 15,336 were identified from dairy farm yard [8], rumen [9], and activated sludge [10], respectively. *L. hyalina* DSM 15,536 was identified as phosphorus removal bacteria because its capability of synthesizing polyphosphate and polyhydroxyalkanoates accumulating bacteria [10]. This suggest *Lampropedia hyalina* might also be resistant to heavy metal stress via polyphosphates-mediated detoxification. Later, researches isolated three other species of *Lampropedia*, including *Lampropedia aestuarii* YIM MLB12 from a sediment sample of the Maliao River estuary [11], *Lampropedia puyangensis* 2-bin from cankered bark tissue of *Populus* × *euramericana* [12], and *Lampropedia cohaerens* CT6 from arsenic rich microbial mats of a Himalayan hot water spring [13]. To note, the draft genome of *L. cohaerens* CT6 also indicated a repertoire of heavy metal resistance genes against arsenic, copper, cobalt, zinc, magnesium, and cadmium [13]. However, to our knowledge, we are currently lack of experimental evidence regarding to heavy metal resistance of *Lampropedia*, and their potential in mixed heavy metal removal remain elusive.

The present study aimed to evaluate the heavy metal resistance and application value of *Lampropedia aestuarii* GYF-1 isolated from soil samples near steel industry. Results demonstrated resistances of *L. aestuarii* GYF-1 to Cd^2+^, Cr^6+^, Cu^2+^, Ni^2+^, Pb^2+^, Zn^2+^ and Fe^2+^ as well as its capability of removing mixed heavy metal contamination. Further integrated genomic analysis and relative gene expression results elucidate adaptive mechanisms for reducing mixed heavy metal stress.

## Results

### Determination of heavy metal in soil samples

We collected total 10 soil samples (0–10 cm) near steel industry in Liuzhou Guangxi Province, where is biggest steel industrial district of south China. The concentrations of heavy metal were evaluated by AAS (Table 1). The results indicated seven heavy metals Cd, Cr, Cu, Ni, Pb, Zn, Fe with average concentration of 1.12, 65.0, 64.0, 44.02, 167.4, 261.52, 280 mg/kg soil, respectively, existed in the samples. We assumed all chromium ions and ferrous ions present in the sample are hexavalent and divalent, respectively, because (1) AAS did not give the valance of information of heavy metals; (2) Cr^6+^ and Fe^2+^ are usually more toxic than Cr^3+^ and Fe^3+^. Thus, a stimulated heavy metal medium (SHMM) containing seven heavy metals (0.2 µM CdCl_2_, 25 µM K_2_CrO_4_, 20 µM CuSO_4_, 15 µM NiCl_2_, 15 µM Pb(CH_3_COO)_2_, 80 µM ZnSO_4_, 100 µM FeSO_4_) was formulated that mimics the heavy metals that present in the soil digested solution (Table 1).

**Table 1:** Measurement of heavy metals in soil sample (n = 10)

Heavy metal	Origin HM conc. (mg/kg soil) n = 10	Stimulated heavy metal medium (µM)
Cd^2+^	1.12	0.2
Cr^6+^	65.00	25
Cu^2+^	64.00	20
Ni^2+^	44.0175	15
Pb^2+^	167.4	15
Zn^2+^	261.52	80
Fe^2+^	280	100

### Isolation of strain GYF-1 from soil sample

**Fig. 1:**
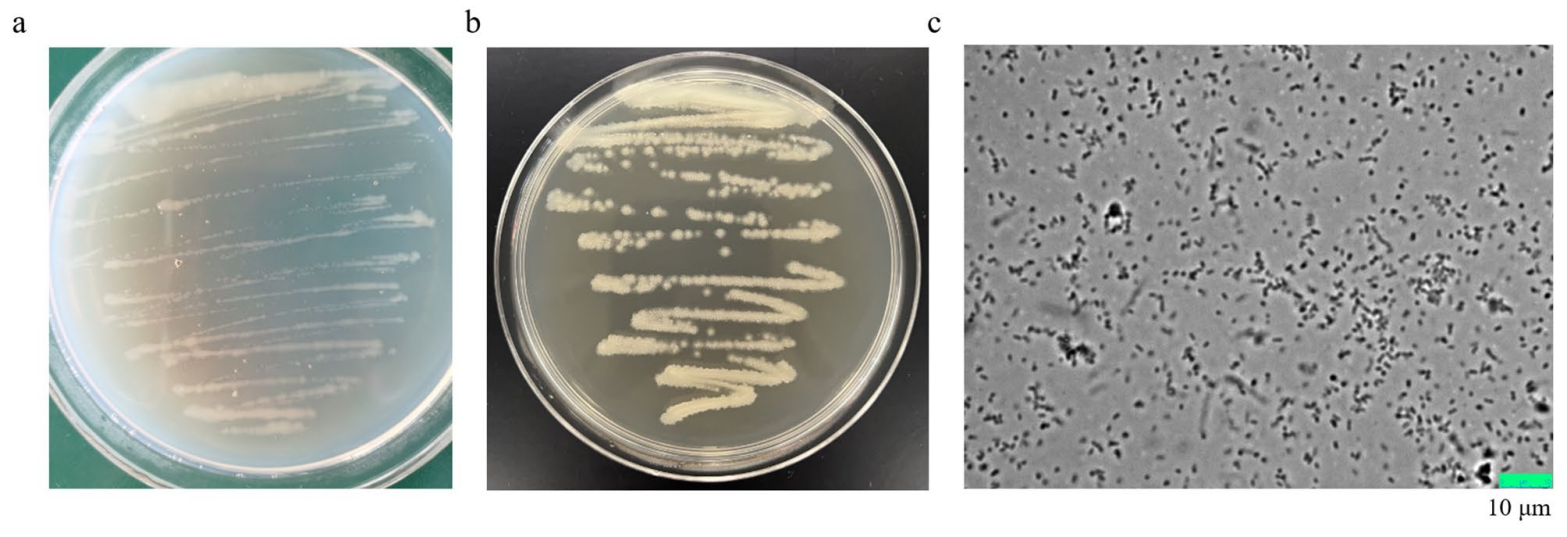
Cell morphology of *L. aestuarii* GYF-1. Colony morphology of *L. aestuarii* GYF-1 on (**A**) LB agar plate and (**B**) TSA plate; (**C**) microphotograph of *L. aestuarii* GYF-1, bar, 10 μm

**Fig. 2:**
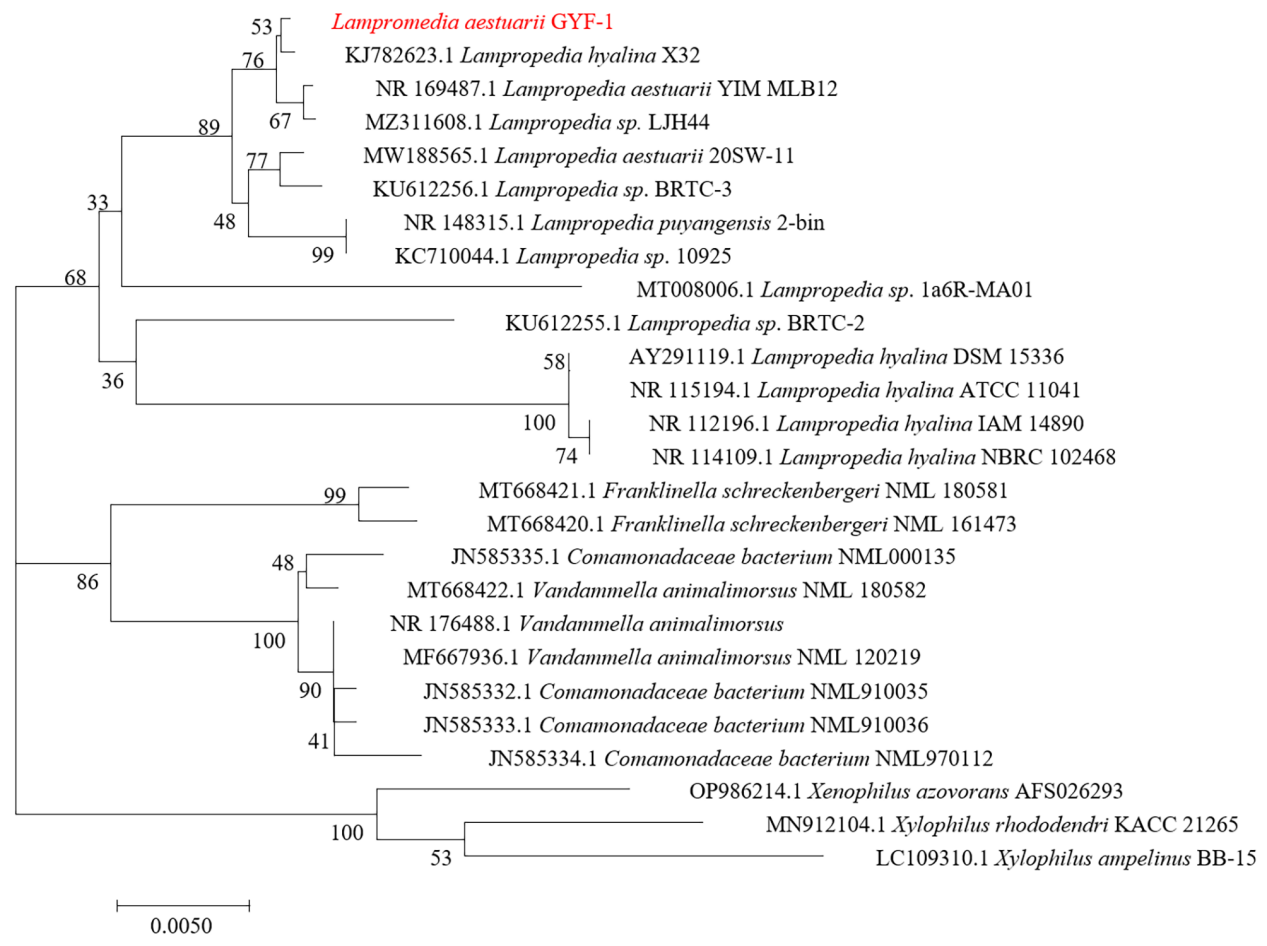
Phylogenetic analysis of *L. aestuarii* GYF-1 based on 16 S rRNA sequences. The 16 S ribosomal RNA sequences were aligned with BioEdit and phylogenetic tree was calculated in MEGA 7.0 by Neighbor-Joining method with bootstrap value of 1000 replicates

Screening of heavy metal resistant bacteria was performed on 0.1 × SHMM agar plate with serially diluted soil suspensions. One of the isolates was identified as *Lampropedia* spp. GYF-1 by 16 S rDNA with identity of 99.92% and 99.84% to *L. hyalina* X32 and *L. aestuarii* YIM MLB12, respectively. GYF-1 colonies were white, circular, opaque, slimy, with ambiguous margin colonies on LB agar (Fig. 1a), and yellow, coarse, wrinkled, with irregular margin on TSA (Fig. 1b), which were different from currently published *Lampropedia* spp. strains. Microscopic check revealed coccoid, unchained cells (Fig. 1c). Strain GYF-1 can grow at a range of temperature from 25^o^C to 37^o^C with optimal of 30^o^C. It could grow in a pH 6.5-8.0, and optimal at pH 7.0. We next performed phylogenetic analysis to gather information on biological diversity and genetic classifications of *Lampropedia* spp. GYF-1. The evolutionary relationship of strain GYF-1 was inferred using Neighbor-Joining based on Kimura 2-parameter method [14]. Strain GYF-1 is phylogenetically close to *L. hyalina* X32, *L. aestuarii* YIM MLB12, and *Lampropedia sp* LJH44 (Fig. 2).

Next, we performed genome analysis (Fig. S1-S5 and Table S1a) and followed by stimulated DNA-DNA hybridization (DDH) by using Genome-to-genome Distance Calculator (GGDC) [15] to identify taxonomy of strain GYF-1 (Table S1b). The draft genome of strain GYF-1 was compared to the genomes of four published *Lampropedia* strains. The results showed strain GYF-1 has close DDH similarity of 87.1% to *L. aestuarii* YIM MLB12 and low similarities to *L. cohaerens* CT6, *L. puyangensis* 2-bin, and *L. hyalina* DSM 16,112, which indicated GFY-1 is a newly discovered *L. aestuarii* strain.

### Heavy metal resistance of strain GYF-1

The resistance of strain GYF-1 to Cd^2+^, Cr^6+^, Cu^2+^, Ni^2+^, Pb^2+^, Zn^2+^, or Fe^2+^, respectively, was determined by minimum inhibition concentration (MIC). The overnight cultured seeds were inoculated to LB medium supplemented with individual heavy metal at OD_600_ of 0.05, and incubated at 30^o^C for 48 h. The MIC values suggested that 2 mM of CdCl_2_, 8 mM of K_2_CrO_4_, 6 mM of CuCl_2_, 4 mM of NiCl_2_, 6 mM of Pb(CH_3_COO)_2_, 0.8 mM of ZnSO_4_, or 4 mM FeCl_2_, respectively, is able to inhibit the growth of strain GYF-1 (Table 2).

**Table 2:** Minimum inhibition concentration of isolate GYF-1

Minimum inhibition concentration of strain GYF-1 in LB medium supplemented with indicated heavy metal (n = 5)														
Heavy metal	0.01mM	0.1mM	0.2mM	0.4mM	0.6mM	0.8mM	1mM	2mM	4mM	6mM	8mM	10mM	0.1 M	MIC (mM)
Cd^2+^	+++	+++	+++	++	++	++	+	+	/	/	/	/	/	2
Cr^6+^	+++	+++	+++	+++	+++	+++	+++	+++	+++	++	+	/	/	8
Cu^2+^	+++	+++	+++	+++	+++	+++	++	++	+	+	/	/	/	6
Ni^2+^	+++	+++	+++	+++	+++	+++	+++	+++	+	/	/	/	/	4
Pb^2+^	+++	+++	+++	+++	+++	+++	+++	+++	++	++	/	/	/	6
Zn^2+^	+++	+++	++	++	+	+	/	/	/	/	/	/	/	0.8
Fe^2+^	+++	+++	+++	+++	+++	+++	+++	+++	+	/	/	/	/	4

‘/’ means OD_600_ < 0.1; ‘+’ means OD_600_ < 0.2; ‘++’ means 0.2 < OD_600_ < 0.4; ‘+++’ means OD_600_ > 0.4, respectively, after 48 h aerobic incubation at 30^o^C. The seeds were inoculated to LB supplemented with indicated heavy metals at OD_600_ of 0.05

### Removal of individual heavy metal

**Fig. 3:**
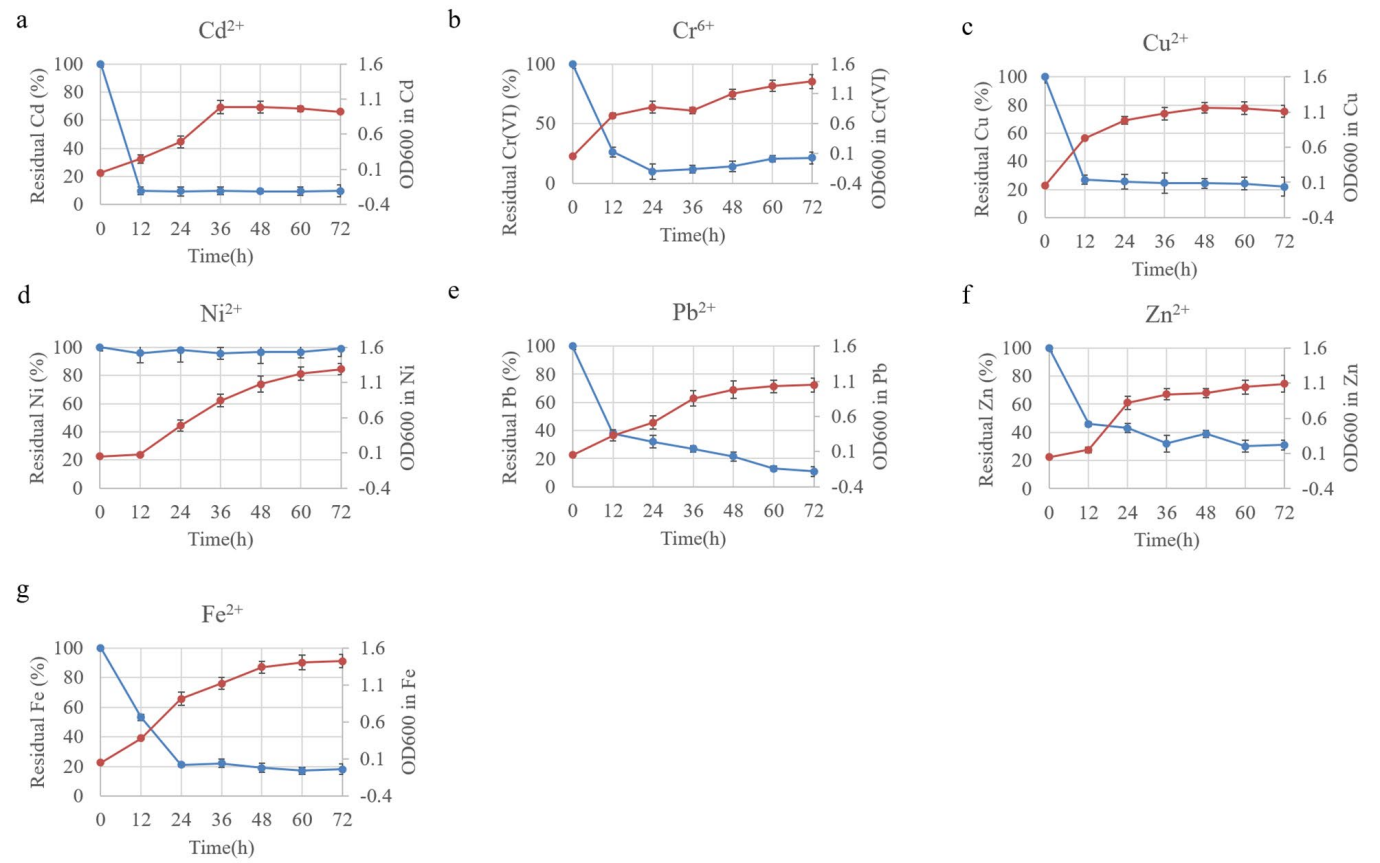
Single heavy metal biosorption of strain GYF-1. The LB medium supplemented with (**A**) 0.8 mM of CdCl_2_, (**B**) 2 mM of K_2_CrO_4_, (**C**) 2 mM of CuCl_2_, (**D**) 2 mM of NiCl_2_, (**E**) 2 mM of Pb(CH_3_COO)_2_, (**F**) 0.6 mM of ZnSO_4_, or (**G**) 2 mM FeCl_2_, respectively, was inoculated with overnight GYF-1 culture broths at OD_600_ of 0.05. The cultures were incubated for 72 h at 30^o^C, and sampled at indicated time for measurement of cell growths and residual heavy metal concentrations by AAS (n = 3)

Next, the metal removal capacity of strain GYF-1 was evaluated. The concentration of individual heavy metal was chosen to be lower than MIC value so that ensure the growth of the strain. Therefore, LB medium supplemented with 0.8 mM of CdCl_2_, 2 mM of K_2_CrO_4_, 2 mM of CuCl_2_, 2 mM of NiCl_2_, 2 mM of Pb(CH_3_COO)_2_, 0.6 mM of ZnSO_4_, or 2 mM FeCl_2_, respectively, was selected (Fig. 3). Strain GYF-1 showed fast removal of Cd^2+^ and Cu^2+^ with removal rate of 90% and 73%, respectively, in 12 h at a relatively low cell density. However, the residual Cd^2+^ and Cu^2+^ maintain at 10% and 25%, respectively, in the medium even if the cell growth continued (Fig, 3a and 3c). Strain GYF-1 reached maximum removal rate of 90% at 24 h, but gradually released 10% of Cr^6+^ into the medium from 24 to 72 h (Fig. 3b). This might be due to the cell death under chromium stress although cell density remain increase. The residual Pb^2+^ and Zn^2+^ decreased very fast with 62% and 54% removal, respectively, at first 12 h, and the removal continued until 72 h at a relatively low adsorption rate (Fig. 3e and f). The rapid removal of Fe^2+^ ended at 48 h with Fe^2+^ concentration of 21% left in the medium, and strain performed a low removal rate from 48 to 72 h even if the cell density reached OD_600_ of 1.4 at final (Fig. 3g). Surprisingly, strain GYF-1 could not remove Ni^2+^ although it grew well as the same in other heavy metals (Fig. 3d), which suggest strain GYF-1 might use efflux system instead of cell surface adsorption or accumulation when nickel ions are present as sole heavy metal stress.

### Removal of mixed heavy metals

**Fig. 4:**
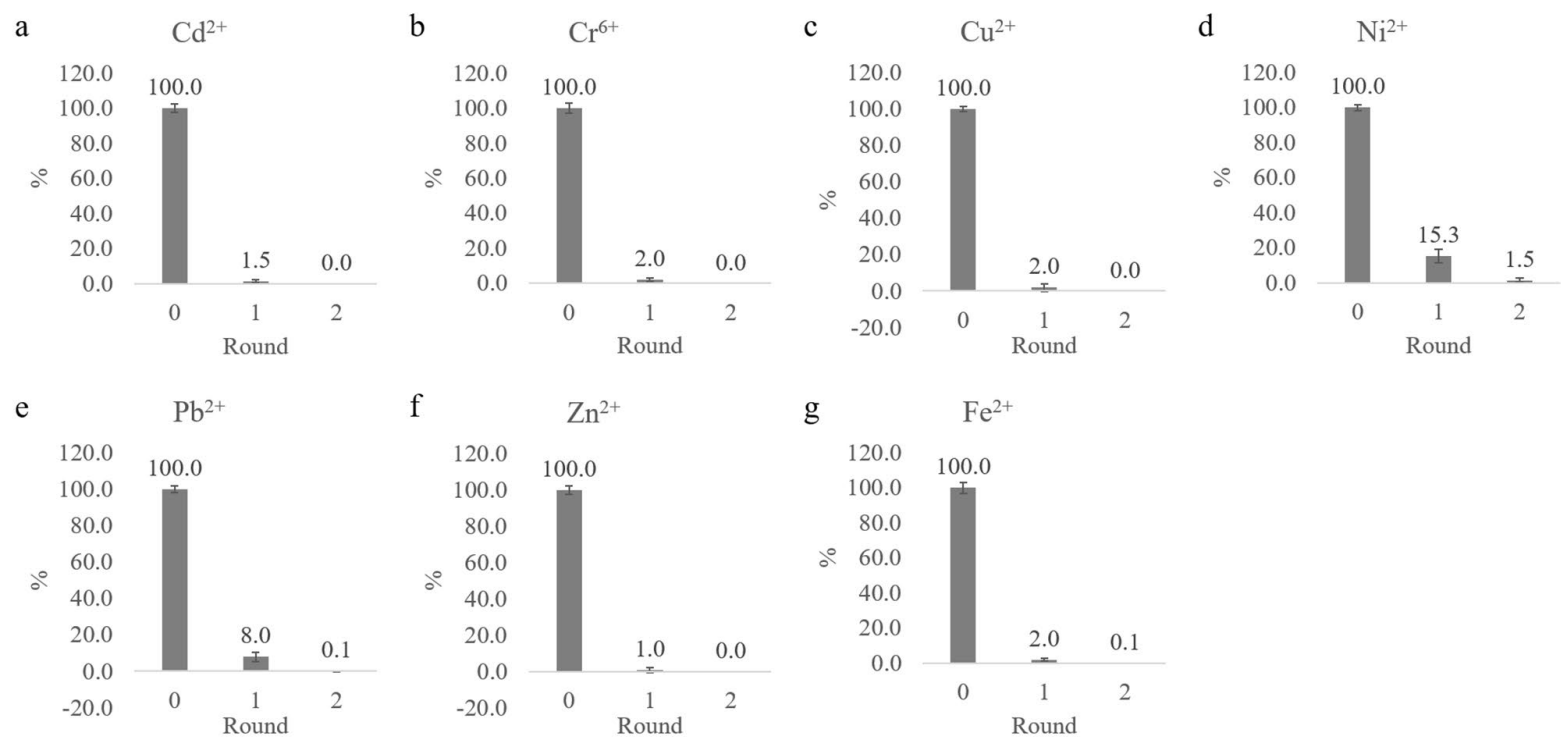
Measurement of residual heavy metal in biosorption assay. Biosorption of mixed heavy metals were using a sequential removal strategy. The 100 ml of soil extract was inoculated with 1 OD_600_ of GYF-1 concentrate, and shaking at 30^o^C for 24 h. The culture was centrifuged, 5 ml of supernatant was sampled, and the rest was inoculated again with GYF-1 concentrate for another 24 h incubation at 30^o^C. The residual concentration of (**A**) Cd^2+^, (**B**) Cr^6+^, (**C**) Cu^2+^, (**D**) Ni^2+^, (**E**) Pb^2+^, (**F**) Zn^2+^, or Fe^2+^ left in all collected samples were determined by AAS (n = 3)

We first evaluated growth of strain GYF-1 in LB medium supplemented with 1 × SHMM. Both specific growth rate and max OD_600_ are impaired in SHMM medium, suggest multiple heavy metal stress (Fig. S6). Considering (1) relatively low cell density of GYF-1 in SHMM medium; and (2) affinity to mixed heavy metals are unknown, we performed two-round sequential removal for soil extract. The first-round removal was able to reduce 98.5%, 98%, 98%, 84.7%, 92%, 99%, and 98% of Cd^2+^, Cr^6+^, Cu^2+^, Ni^2+^, Pb^2+^, Zn^2+^ and Fe^2+^, respectively, suggest still high amount of Ni^2+^ and Pb^2+^ ions left (Fig. 4). After second-round removal, the levels of Cd^2+^, Cr^6+^, Cu^2+^, and Zn^2+^ were under detection, and residual concentrations of Ni^2+^, Pb^2+^ and Fe^2+^ were in accepted levels, respectively. Interestingly, strain GYF-1 did not show nickel removal when Ni^2+^ solely presented in the medium (Fig. 3d); however, GYF-1 was able to remove Ni^2+^ ions in mixed heavy metal stress (Fig. 4d). One explanation is that nickel resistance of GYF-1 might efflux mediated but with no adsorption of the nickel ions, which agreed with that many nickel transporters are annotated on the genome (Table S4). On contrary, the EPS triggered by other heavy metals could also interact with Ni^2+^ ions in mixed heavy metal condition.

### Lipopolysaccharides contributes to mixed heavy metal stress adaptation

Next, the adaptive mechanisms used by *L. aestuarii* GYF-1 to mixed heavy metal stress were evaluated. Lipopolysaccharides is the major outer surface in most Gram-negative bacteria [16]. Annotations of *L. aestuarii* GYF-1 genome indicated genes involve in lipopolysaccharide biosynthesis (Table 3). However, genes encoding biosynthesis of an inner core with three L-glycero-*D-manno*-heptose sugars (HepI, HepII, and HepIII) are missing, which probably because a few sequence gaps are missing in the *L. aestuarii* GYF-1 genome.

**Table 3:** Predicted genes in LPS biosynthesis

Gene Tag	Gene	Annotation
KDO biosynthesis		
GYF_00028	*kdsA*	3-deoxy-D-manno-octulosonic acid (KDO) 8-phosphate synthase
GYF_02650	*kdsB*	3-deoxy-manno-octulosonate cytidylyltransferase
GYF_02059	*kdsC*	3-deoxy-D-manno-octulosonate 8-phosphate phosphatase
GYF_02058	*kdsD*	arabinose-5-phosphate isomerase
GYF_03747	*atoA*	3-deoxy-D-manno-octulosonate 8-phosphate phosphatase
GYF_03746	*atoD*	Arabinose-5-phosphate isomerase
Lipid A biosynthesis		
GYF_01039	*lpxA*	UDP-N-acetylglucosamine acyltransferase
GYF_01040	*lpxB*	Lipid A disaccharide synthetase
GYF_01706	*lpxC*	UDP-3-O-[3-hydroxymyristoyl] N-acetylglucosamine deacetylase
GYF_01037	*lpxD*	UDP-3-O-[3-hydroxymyristoyl] glucosamine N-acyltransferase
GYF_01102	*lpxH*	UDP-2,3-diacylglucosamine hydrolase
GYF_02844	*lpxK*	Tetraacyldisaccharide-1-P 4’-kinase
GYF_03243	*lpxL-1*	Lipid A biosynthesis lauroyl acyltransferase
GYF_03244	*lpxL-2*	Lipid A biosynthesis lauroyl acyltransferase
Core oligosaccharide biosynthesis		
GYF_03850	*rfaG*	Glycosyltransferase
GYF_02341	*rfaL*	O-Antigen ligase
GYF_02561	*rfaS*	Lipopolysaccharide core biosynthesis protein
GYF_00179	*kdtA*	3-deoxy-D-manno-octulosonic-acid transferase
GYF_02340	*wcaA*	UDP-glucose LOS-beta-1,4 glucosyltransferase
O-polysaccharide receptor biosynthesis		
GYF_03348	*wbbL*	Rhamnosyltransferase
GYF_00472	*rmlA1*	Glucose-1-phosphate thymidylyltransferase 1
GYF_00473	*rmlC*	dTDP-4-dehydrorhamnose 3,5-epimerase
GYF_02287	*rmlD*	dTDP-4-dehydrorhamnose reductase
GYF_02289	*rmlB*	dTDP-glucose 4,6-dehydratase
Lipid A modification		
GYF_03803	*eptA*	Lipid A phosphoethanolamine transferase
LPS transport		
GYF_00897	*lptA*	Lipopolysaccharide transport periplasmic protein
GYF_00165	*lptD*	Lipopolysaccharide transport protein D
GYF_00912	*lptG*	LPS export ABC transporter permease

**Fig. 5:**
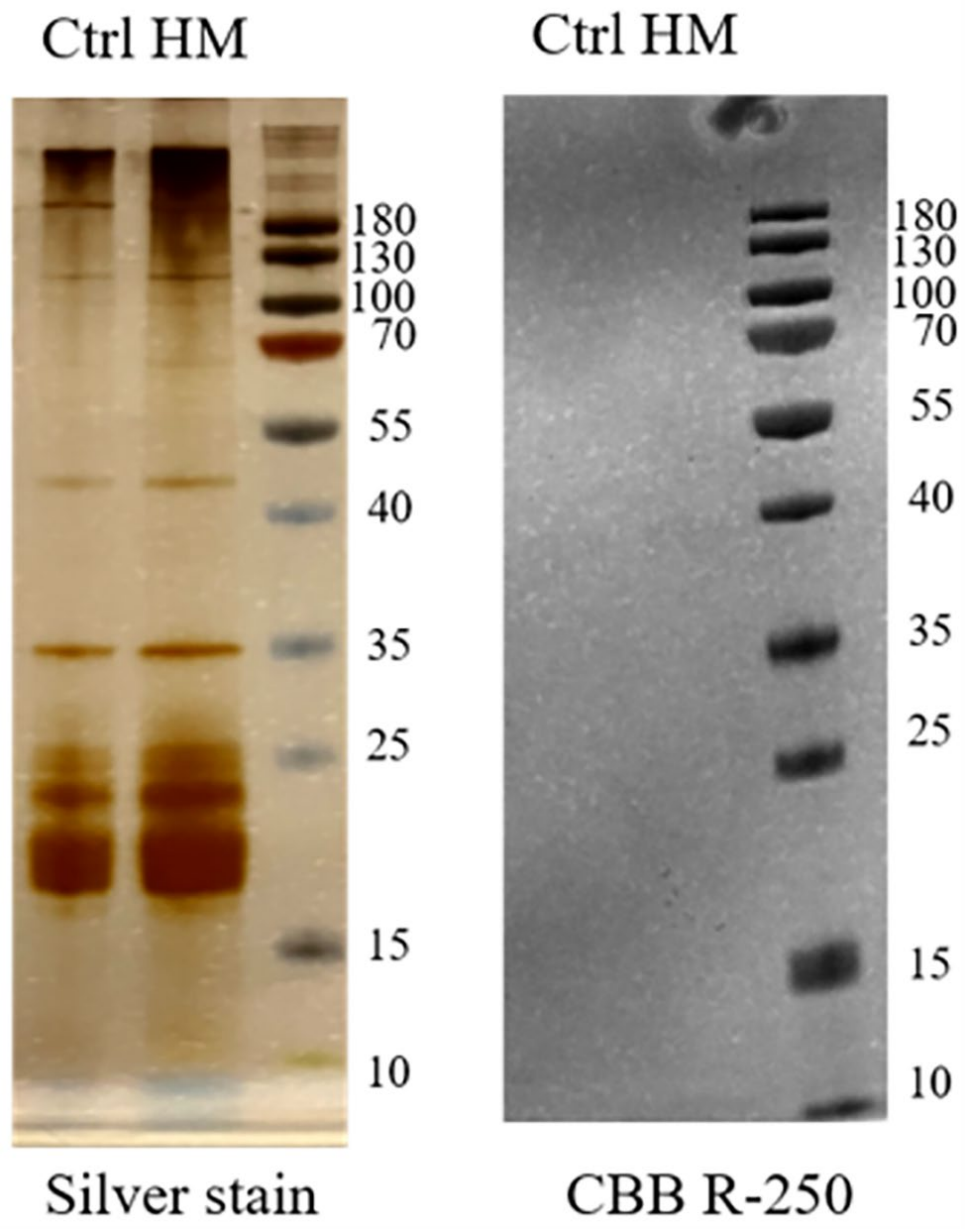
Evaluation of lipopolysaccharides in mixed heavy metal stress. LPS amounts and biofilm formation were evaluated under control and mixed heavy metals conditions. Silver staining (left) of extracted LPS, and coomassie blue R-250 staining (right) used to examine protein contamination. 10 mg of cell pellets were subjected to LPS extraction with two biological replicates, 10 µL of each sample was loaded to each lane for relative LPS level comparison

**Fig. 6:**
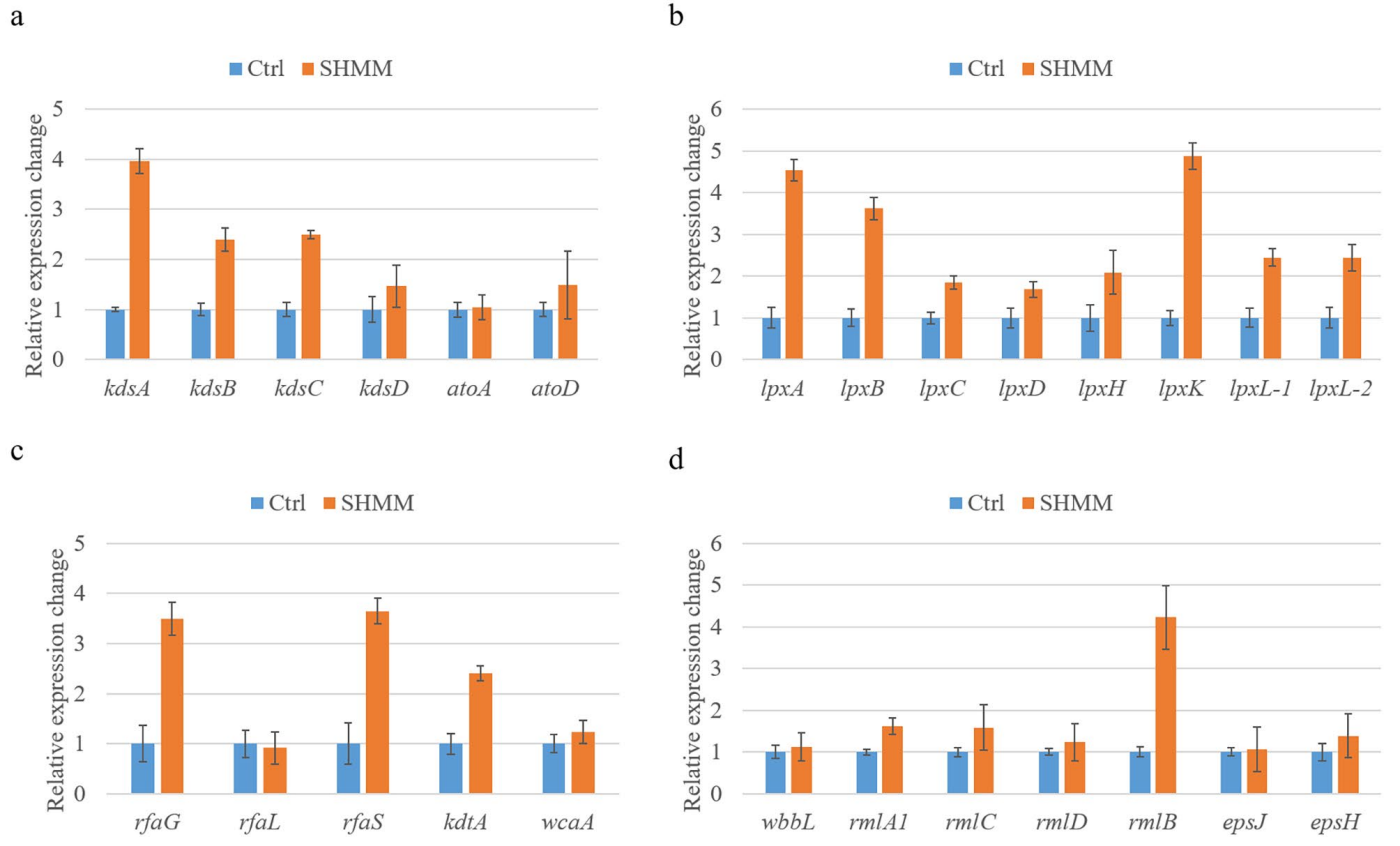
Relative expressions of LPS synthesis genes response to mixed heavy metals. Relative gene expressions involved in (**a**) KDO biosynthesis; (**b**) lipid A biosynthesis; (**c**) core oligosaccharide biosynthesis; (**d**) O-polysaccharide receptor biosynthesis. Bars represented the mean of three technical replicates and the error bars the standard error of the mean (mean ± SEM, n = 3)

Gram-negative bacteria usually express LPS consists of O-antigen, complete core oligosaccharides, and the lipid A. Previous research indicated some bacteria modify LPS amount or structure to adaptively respond to metal stress [17]. To examine if *L. aestuarii* GYF-1 also use this strategy to possess mixed heavy metal adaptation, we first compared LPS level in between control and mixed heavy metal condition (Fig. 5). To examine if *L. aestuarii* GYF-1 also use this strategy to possess mixed heavy metal adaptation, we first compared LPS level in between control and mixed heavy metal condition (Fig. 5). The silver staining result indicated significantly increased level of LPS in mixed heavy metals, but the band patterns of the LPS were similar in both conditions, suggest mixed heavy metal adaptation was mainly through adjustment of LPS level. Next, we compared relative expression level of genes involve in LPS biosynthesis. As expected, expressions of four genes *kdsA* (GYF_00028), *kdsB* (GYF_02650), *kdsC* (GYF_02059) *kdsD* (GYF_02058) for 3-deoxy-D-manno-octulosonic acid (KDO) biosynthesis are upregulated under mixed heavy metal stress (Fig. 6a). To note, GYF_03746 and GYF_03747 were predicted as arabinose-5-phosphate isomerase and 3-deoxy-D-manno-octulosonate 8-phosphate phosphatase, respectively; however, they were not up-regulated under stress condition. For Lipid A biosynthesis, the relative expressions of all detected genes were significantly increased in mixed heavy metal stress (Fig. 6b). The *rgaG*, *rfaS*, and *kdtA* involved in core oligosaccharide biosynthesis were at least 2-fold increased (Fig. 6c). Taken together, these up-regulated gene expressions indicated the requirement of core oligosaccharide and lipid A in mixed heavy metal adaptation.

O-antigen is a polymer with highly variable oligosaccharide repeating subunits at the most external portion of the LPS [16]. Annotations of *L. aestuarii* GYF-1 genome reveals the presence of four genes involve in L-rhamnose biosynthesis which is an important component of O- polysaccharide [18]. We noticed most of genes involved in O-polysaccharide receptor and polysaccharide biosynthesis were not up-regulated under mixed heavy metal stress, except for dTDP-glucose 4,6-dehydratase *rfbB* (GYF_02289) (Fig. 6d), suggest the regulation of O-polysaccharide biosynthesis was not mainly at transcriptional level in mixed heavy metal stress.

On the other hand, the up-regulation of the KDO and lipid A biosynthesis was consistent with the higher relatively expressions of components in Type II and Type III secretion pathway, which are probably required for exportation of LPS synthesis-related enzymes (Fig. S8 and Table S3). Taken together, these suggest *L. aestuarii* GYF-1 increased level of lipopolysaccharides and to adaptively respond to mixed heavy metal stress.

### Biofilm formation involves in mixed heavy metals adaptation

**Fig. 7:**
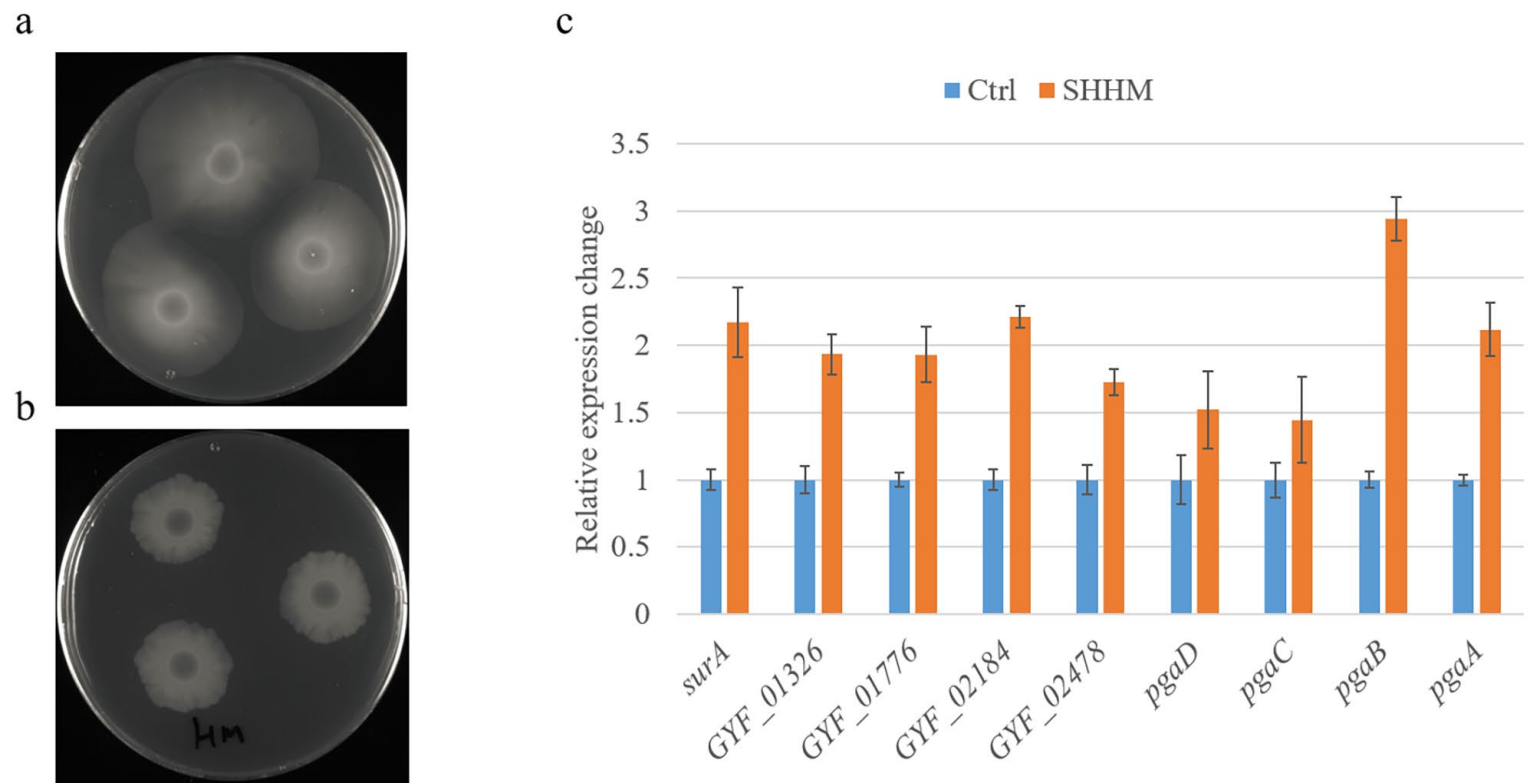
Evaluation of biofilm formation in mixed heavy metal stress. (**a** and **b**) cell morphologies on (**a**) LB agar and (**b**) LB agar supplemented with SHMM (n = 5). (**c**) Relative gene expressions involved in biofilm formation. Bars represented the mean of three technical replicates and the error bars the standard error of the mean (mean ± SEM, n = 3)

LPS was involved in biofilm formation [19]. To verify our hypothesis if biofilm formation involves in mixed heavy metal adaptation, we first checked the cell morphology in SHMM agar plate. *L. aestuarii* GYF-1 formed clear, thick, and tight biofilm under mixed heavy metal stress compared to the vague, smooth, and loosen shape under normal LB growth (Fig. 7a and b). Second, the relative expression levels with regards to biofilm formation were checked in liquid 1 × SHMM culture (Fig. 7c). As expected, three annotated positive transcriptional regulators, GYF_01326, GYF_02184, GYF_02478, and peptidylprolyl isomerase encoding gene GYF_01776 were all up-regulated. The linear homopolymer poly-β-1,6-N-acetyl-d-glucosamine (PGA) are the gene product of the *pgaABCD* operon and functions as an adhesin for the maintenance of biofilm structural stability [20]. Results indicated expressions of *pgaA* (PGA export porin) and *pgaB* (PGA N-deacetylase) were at least 2-fold higher in mixed heavy metal stress that in normal growth; the expressions of two PGA synthase, *pgaC* and *pgaD*, were slightly increased but to a lesser extent. In addition, the up-regulated secretion pathway might also contribute to biofilm formation (Fig. S8). Taken together, these results indicated biofilm formation as an adaptive method used by *L. aestuarii* GYF-1 for mixed heavy metal detoxification.

## Discussions

The present study isolated one heavy metal-resistant *L. aestuarii* strain from soil samples near steel factory and demonstrated its potentiality in biosorption of mixed heavy metals. Currently, the *Lampropedia* genus is represented by four species, *L. hyalina*, *L. puyangensis*, *L. cohaerens*, and *L. aestuarii*, however, their heavy metal resistance and potential in application remain elusive. Here, we experimentally demonstrated *L. aestuarii* GYF-1 is resistance to multiple heavy metal stress with increased LPS level and biofilm formation.

*L. aestuarii* GYF-1 was isolated from soil samples containing heavy metals, which suggest strain GYF-1 have developed adaptation mechanism to mixed heavy metal stress. The annotations revealed a repertoire of metal resistance genes in *L. aestuarii* GYF-1 genome (Table S4), suggest they might be responsible for efflux of the heavy metals. Bacterial chromium resistance usually via chromium reductase encoded by chromium reductase and efflux system, which involves chromium transporter encoded by *chrA* and a *chrA* positive regulator *chrB* [21]. The predictions of Cr-resistance genes indicated one gene GYF_02978 encodes chromate transporter gene *chrA* in Scaffold 5, however, no *chrB* gene was identified, suggest the *ChrA* functions alone in strain GYF-1. Two cupin-like domain transcription activator genes *chrR* were found in Scaffold 1 and Scaffold 9. For copper resistance, *yfiH* (GYF_00340) encodes multicopper polyphenol oxidoreductase laccase was found in Scaffold 1. The *copZ* is a known encodes cytoplasmic copper chaperone, which was predicted in Scaffold 13 (GYF_00913). A MerR-family transcription factor *cueR* was predicted in Scaffold 8 (GYF_03485). In addition, GYF_01534 was predicted as a potential copper(I)-binding protein although its function was not annotated in *Lampropedia* spp. Analysis of nickel transporter predicted many genes encode *nikABCDE* transporter in GYF-1 genome. This could explain previous finding that strain GYF-1 have high nickel tolerance but with no adsorption in nickel stress, because the main mechanism of nickel resistance used by GYF-1 is via efflux mediated by transporters. A whole *nikABCDE* gene cluster (GYF_01655–01659) was annotated in Scaffold 2, and partial *nik* gene clusters were predicted in Scaffold 3, 8, 11, 16, 20, suggest the *nik* transporter complex in GYF-1 might be different with those classic systems reported in model bacteria. Besides those single heavy metal specific transporter, many multiple heavy metal transporters were also annotated. The *czcABC* gene cluster (GYF_02595–02597) was annotated in Scaffold 4, which encode heavy metal efflux pump of cobalt, zinc, and cadmium. Two P1B-type ATPase genes *zntA* (GYF_03486 and GYF_03587) which confer resistance specifically to Pb^2+^, Zn^2+^, and Cd^2+^ were found in Scaffold 8. The ABC-type Mn^2+^/Zn^2+^ transport system *znu* was predicted in Scaffold 5, with two *znuB* (GYF_02916 and GYF_02917) and one *znuC* (GYF_02918), suggest the difference of *znuABC* complex in *L. aestuarii* GYF-1 from other studied species. The presence of multiple efflux transporters could also explain *L aestuarii* GYF-1 exhibited high tolerance to individual heavy metal stress as indicated by MIC values (Table 2). To note, the mechanism that bacterial responses to individual heavy metal or mixed stress might be different. Therefore, the role of these transporters in heavy metal stress needs to be further validated.

Polyphosphates (polyP), as polyanions, are involved in detoxification of heavy metals [22]. Annotation revealed two polyphosphate kinase encoding genes, *ppk1* (GYF_02745) and *ppk2* (GYF_02770), are involved in polyphosphate metabolic process. The expression of *ppk1* and *ppk2* was assessed to evaluate if inorganic polyP contributes to intracellular heavy metal sequestration. The expression of *ppk1* was 2.18-fold higher in 1 × SHMM than in control medium, and no significant up-regulation was found with regards to *ppk2* expression (Fig. S9). This indicated the polyP might contribute to mitigation of mixed heavy metal stress, but might not play the major role for biosorption. One explanation is that the intracellular compartment depends on the concentration of heavy metals inside the cells. In addition, genome annotation also predicted the presence of lipoic acid biosynthesis pathway [23] and superoxide dismutase (SOD) [24], which are important to alleviate reactive oxygen stress generated by heavy metals.

Biosorption is defined as adsorption of substances by using passive physiochemical pathways, such as electrostatic forces and ion/proton displacement, while bioaccumulation is active metabolic event in which heavy metals are taken up into the cell [5]. In this study, our current finding suggested detoxification of mixed heavy metal stress mainly through biosorption. For bioaccumulation, genome annotations indicated the presence of two proteins containing heavy metal binding motif (Table S1). GYF_02298 was annotated as a thiol-disulfide isomerase containing CXXC motif which is responsible for multi heavy metal binding [25]. GYF_03532 was predicted as a heavy metal sensor kinase containing a sensor histidine kinase domain [26]. However, if these two proteins actively contribute to bioaccumulation of heavy metal remain elusive.

For biosorption application, we applied two-round adsorption strategy into removal of mixed heavy metals pollution. *L. aestuarii* GYF-1 showed biosorption efficiency of greater than 90% in removing Cd^2+^, Cr^6+^, and Pb^2+^, however, relatively low efficiency in adsorbing Cu^2+^ and Zn^2+^ and no affinity to Ni^2+^, individually. The biosorption efficiency is impacted by various factors, such as pH, temperature, initial concentration of the heavy metals, cell density, treatment time, etc [5]. Thus, optimization of adsorption condition might be necessary to improve the biosorption efficiency. On the other hand, mixed heavy metal biosorption assay proved the concept that *L. aestuarii* GYF-1 could be used as biosorbent for bioremediation of soil samples in a lab-scale. Several bioprocess factors also should be taken into considerations when scale-up to industrial level, for example type of bioreactor, pH, and temperature control, mixing and agitation, feeding strategy (in batches or in continuous mode) [27, 28].

## Conclusions

In this study, we isolated a mixed heavy metal resistant *L. aestuarii* strain from soil samples near steel factory. Heavy metal tolerance assay indicated *L. aestuarii* GYF-1 possessed MIC value of 2 mM, 10 mM, 6 mM, 4 mM, 6 mM, 0.8 mM, and 4 mM for CdCl_2_, K_2_CrO_4_, CuCl_2_, NiCl_2_, Pb(CH_3_COO)_2_, ZnSO_4_, and FeCl_2_, respectively. The biosorption assay demonstrated its capacity in bioremediation of soil polluted by mixed heavy metals. Genome analysis revealed abundance of heavy metal resistance genes in the genome of *L. aestuarii* GYF-1. Further evaluation on differential gene expressions under stress condition suggest enhanced LPS and biofilm formation contributed to the adaptation of mixed heavy metals. This study demonstrated *L. aestuarii* GYF-1 can be selected as a potential candidate strain for biosorption application.

## Materials and methods

### Soil sample collection and analysis

For soil sample collection, 10–20 g soils a depth of 10 cm below the surface, and a total of 10 samples were collected using an ordinary shovel. The sampling site was 3-km away from a steel factory in Liuzhou, Guangxi Province, South China. Each sampling site was spaced at least 500 m apart.

To determine the heavy metal concentration, the collected sludge samples were treated according to GB 15,618 − 2018 [29]. Briefly, the soil samples were air-dried and sieved. Then, 0.5 g of dry soil were digested with an acid mixture (hydrofluoric acid-nitric acid-hydrochloric acid). The digested solution was cooled, filtered, and finally diluted to 25 mL. The concentrations of heavy metal were determined by atomic absorption spectroscopy (AAS). The original concentration of Cd, Cr, Cu, Ni, Pb, Zn, and Fe ions were 1.1 ± 1.0, 65 ± 17, 64 ± 23, 44 ± 18, 167 ± 42, 262 ± 26, and 280 ± 65 mg/kg soil, respectively. Hence, the concentration of Cd, Cr, Cu, Ni, Pb, Zn, and Fe ions in digested solution were 0.2 ± 0.18, 25 ± 6.54, 20.14 ± 7.24, 15.52 ± 6.35, 16.12 ± 4.05, 80.15 ± 7.95, and 100.27 ± 23.28 µM, respectively.

### Media and strain isolation

A stimulated heavy metal medium (SHMM) containing seven heavy metals was formulated that mimics the heavy metals that present in the soil digested solution. Thus, 1 × SHMM contains basal LB medium and supplemented with 0.2 µM CdCl_2_, 25 µM K_2_CrO_4_, 20 µM CuSO_4_, 15 µM NiCl_2_, 15 µM Pb(CH_3_COO)_2_, 80 µM ZnSO_4_, and 100 µM FeSO_4_.

For isolation of heavy metal resistant bacteria, 0.5 g of sieved soil sample was mixed with 25 ml sterile ddH_2_O, vortexed at room temperature (RT) for 10 min. One microliter of suspension was used for 10-fold serial dilution, and spread 1 ml of each diluted gradients on 0.1 × SHMM agar plate. The plates were aerobically incubated at either 30^o^C or 37^o^C for 48 h, and then individual colony was purified on 0.1 × SHMM agar plate.

### Atomic absorption spectroscopy

The biosorption of heavy metals was determined by using atomic absorption spectroscopy. The overnight cultured bacteria were inoculated in 100 mL of LB broth supplemented with individual heavy metal or with 1 × SHMM at 30^o^C for indicated time. The samples were collected, cell pellets were removed by centrifugation at 7,000 rpm for 5 min. The supernatant was digested using HNO_3_ to oxidize to a single high-valence state or converted into inorganic compounds, and digest was diluted to an appropriate concentration. The metal concentrations were determined by using Agilent AA 240FS (Agilent). The parameters including air flow, acetylene flow, characteristic wavelength, slit width, lamp current were according to the manufacturer’s instructions. The removal of heavy metal was equal to the initial concentration of heavy metal in the medium subtracted by the residual heavy metal concentration in the supernatant.

### Identification of isolate GYF-1

The isolate was cultured in Luria-Bertani (LB) medium to observe the colony morphology, and cell shape was microscopically checked. Colony PCR was directly used for amplification of 16S rDNA by universal primers (8F: 5’-AGA GTT TGA TCC TGG CTC AG-3’ and 1492R: 5’-CGG TTA CCT TGT TAC GAC TT-3’). The PCR fragments were electrophorized, sent for Sanger sequencing (Sangon, Shanghai), and blasted on NCBI website. For phylogenetic analysis, the 16 S rDNA sequences of close bacterial strains were clustered by ClustalW [30], and the phylogenetic tree was constructed through Neighbor-Joining method by MEGA 7 [31].

### Genome analysis of *L. aestuarii* GYF-1

The genomic DNA of GYF-1 was prepared by using Quick-DNA Kits (ZymoResearch). The genomic DNA was quantified by Qubit dsDNA HS Assay Kit (ThermoFisher). The 500 ng of genomic DNA was used for DNA library preparation by using NEB Next Ultra DNA Library Prep Kit for Illumina. The quality of the DNA library with length of ~ 500 bp was electrophoresed and quantified by Thermo Qubit 4.0 (ThermoFisher). Sequencing was performed on Illumina HiSeq 4000 platform with 150 bp paired-end mode.

The acquired sequence reads were subjected to quality filtering and de novo assembly using SPAdes 3.5.0 [32]. The gaps were closed by GapFiller 1.11 [33], and sequence correction was done by PrInSeS-G 1.0.0 [34]. The *in silico* DNA-DNA hybridization (DDH) using Genome-to-Genome Distance Calculator (GGDC) [15] was performed to distinguish isolate GYF-1 at species level.

For genome annotation, the coding genes and non-coding RNAs were predicted by Prokka 1.10 [35], interspersed repeats were screened by RepeatMasker [36]. Annotation was performed with the Cluster of Orthologous Groups of proteins (COG) [37], SwissProt [38], TrEMBL [39], Protein family (PFAM) [40], Conserved Domain Database (CDD) [41], Kyoto Encyclopedia of Genes and Genomes (KEGG) database [42], and NR database [43] through a BLAST + search. Gene Ontology (GO) annotation [44] was based on results from SwissProt and TrEMBL.

### Heavy metal tolerance of *L. aestuarii* GYF-1

The minimum inhibitory concentration (MIC) values of *L. aestuarii* GYF-1 were tested for heavy metals, including CdCl_2_, CrCl_3_, K_2_CrO_4_, CuSO_4_, NiCl_2_, Pb(CH_3_COO)_2_, and ZnSO_4_. The LB medium supplemented with 0.1 M of individual heavy metal (pH was adjusted either by NaOH or HCl to 6.8) was diluted to indicated concentration. The overnight culture broth was inoculated to final OD_600_ of 0.05. The strain was considered tolerant to a particular heavy metal concentration if OD_600_ higher than 0.2 at 30 °C for 48 h incubation. The MIC was defined as the lowest concentration of a heavy metal inhibiting bacterial growth for 2 days.

### Single heavy metal removal assay

For evaluating the biosorption capacity of *L. aestuarii* GYF-1, LB medium was supplemented with 0.8 mM CdCl_2_, 2 mM CrCl_3_, 2mM K_2_CrO_4_, 2mM CuSO_4_, 2mM NiCl_2_, 6 mM Pb(CH_3_COO)_2_, or 0.6 mM ZnSO_4_, respectively. The pH of the LB-heavy metal medium was adjusted to 6.8 either by NaOH or HCl. For biosorption assay, 100 ml of medium was inoculated with overnight culture to OD_600_ of 0.05, and incubated at 30^o^C with shaking. Every 12 h, 5 ml culture was sampled for measurement of OD_600_ and residual heavy metal concentration by AAS. The removal rate of individual heavy metal in the sample was calculated by Eq. 1.

$${\rm{Heavy}}\,{\rm{metal}}\,{\rm{removal}}\,\left( {\rm{\% }} \right)\,{\rm{ = }}\,\left( {{{\rm{C}}_{{\rm{init}}}}{\rm{ - }}\,{{\rm{C}}_{{\rm{sample}}}}} \right)\,{\rm{/}}\,{{\rm{C}}_{{\rm{init}}}}{\rm{ \times }}\,{\rm{100}}$$

where C_init_ is the initial concentration of heavy metals in the medium; C_sample_ is the concentration of heavy metal in collected sample.

### Mixed heavy metal removal assay

The survival of *L. aestuarii* GYF-1 in mixed heavy metal stress was firstly assessed in 1 × SHMM that mimics the heavy metals that present in the soil digested solution. The overnight culture broths were inoculated at OD_600_ of 0.05, and cultured at 30^o^C for 72 h. To prepare the GYF-1 concentrate used for bioremediation, the culture broth was first prepared in LB supplemented with 1 × SHMM for 24 h at 30^o^C, the cell suspension was washed twice by PBS, concentrated by centrifuge, and kept at 4^o^C for further use.

Removal of mixed heavy metals were using a sequential removal strategy. The soil samples were air-dried and sieved. Then, 2 g of dry soil were digested with an acid mixture (hydrofluoric acid-nitric acid-hydrochloric acid). The digested solution was cooled, filtered, adjusted pH to 6.8 by NaOH and diluted to 50 mL, and then 50 mL 2 × LB medium was added for preparation of soil extract. Five milliliter of soil extracts were sampled to determine the initial heavy metal concentrations.

In first-round remediation, 1 OD_600_ of GYF-1 concentrate was inoculated to 100 mL of soil extract, and shaking at 30^o^C for 24 h. The culture was centrifuged, 5 ml of supernatant was sampled, and the rest was inoculated again with GYF-1 concentrate in the same growth condition as second-round adsorption. The removal of heavy metals in collected samples were determined by AAS and calculated by Eq. 1.

### Extraction of LPS

Extraction of LPS was using hot phenol-water method as described previously with some modifications [45]. Briefly, 10 mg of wet cell pellets were collected by centrifuge, washed twice with PBS containing 0.15 mM CaCl_2_ and 0.5 mM MgCl_2_. Pellets were then resuspended in PBS, and sonicated for 10 min on ice. To eliminate the contamination of proteins and nucleic acids, 10 µg/mL of proteinase K (Roche) was added to the cell mixtures and tubes were kept at 65^o^C for 1 h, the mixtures were subsequently treated with 20 µg/mL of DNase I (ThermoFisher), 40 µg/mL of RNase (Sangon, Shanghai) at 37 ^o^C overnight. Next, an equal volume of 90% hot phenol (65^o^C) was added to the mixtures, vortexed vigorously, and incubated at 65^o^C for 15 min. The mixtures were cooled down on ice, and centrifuged at 12,000 rpm for 10 min at 4^o^C, supernatants were transfer to new tubes, and phenol phases were re-extracted with 100 µL of water twice. The upper aqueous phase from each step were combined. The crude extracts were further precipitated by two volume of 0.375 M MgCl_2_ in 95% ethanol at -20^o^C. After the samples had cooled to 0^o^C, they were centrifuged at 12,000 rpm for 15 min at 4^o^C. The pellets were re-suspended in 1 × sampling buffer, boiled at 70^o^C for 5 min, electrophoresed by using NuPAGE 12% Bis-Tris Gel system (ThermoFisher), and stained by either silver staining by Mass Silver Stain kit (Sangon, Shanghai) or coomassie blue R-250 (Sangon, Shanghai) according to the manufacturer’s instructions.

### RNA extraction

Overnight culture of strain GYF-1 was inoculated to LB with and without supplemented with 1 × SHMM at final OD_600_ of 0.05. The cultures were incubated for 24 h at 30^o^C at 200 rpm, and harvest by centrifugation at 4,000 rpm at 4^o^C for 10 min, and washed once by cold 1 × TE (10 mM Tris-HCl, 1 mM EDTA, pH 7.8). Extraction of total RNA was performed by using Bacteria Total RNA Isolation Kit (Sangon, Shanghai) following the manufacturer’s instruction. The resuspended RNA was treated with DNase I (ThermoFisher), and purified with the RNA Clean & Concentrator kit (ZymoResearch). The resulting RNA concentration was measured with a Biospectrometer (Eppendorf).

### cDNA synthesis and qRT-PCR

cDNA synthesis was performed using the PrimeScript RT Reagent Kit (Takara) according to the manufacturer’s protocol with 1 µg of total RNA as template, and aliquot without adding PrimeScript RTase with DEPC-treated ddH_2_O was used as no-RT negative control to evaluate genomic DNA contamination. Primers used for qPCR were designed using Primer designing tool and list of primers was available (Table S3). The qPCR was performed using a StepOnePlus Real-Time PCR System (ThermoFisher) and the reaction mixtures were prepared in triplicate for each sample using the TB Green Premix Ex Taq (Takara). The 16 S rRNA gene was used as housekeeping gene reference. Changes in transcript abundance were automatically calculated using the ΔΔC_*T*_ method.

### Electronic supplementary material

Below is the link to the electronic supplementary material.

Supplementary Material 1

Supplementary Material 2

Supplementary Material 3

Supplementary Material 4

Supplementary Material 5

## Data Availability

Genome of *L. aestuarii* GYF-1 are available on NCBI (https://www.ncbi.nlm.nih.gov/) with accession number PRJNA958147. Results of the genome annotations and primers are available in the supplementary materials.
