# Supplementary material for: Isolation of a novel multiple-heavy metal resistant Lampropedia aestuarii GYF-1 and investigation of its bioremediation potential
Source: BMC Microbiol. 2023 Nov 7;23:330. doi: 10.1186/s12866-023-03093-4 (PMC10629017; doi:10.1186/s12866-023-03093-4)
Supplement: Supplementary file 5 — Supplementary Material 5 [file 12866_2023_3093_MOESM5_ESM.docx]

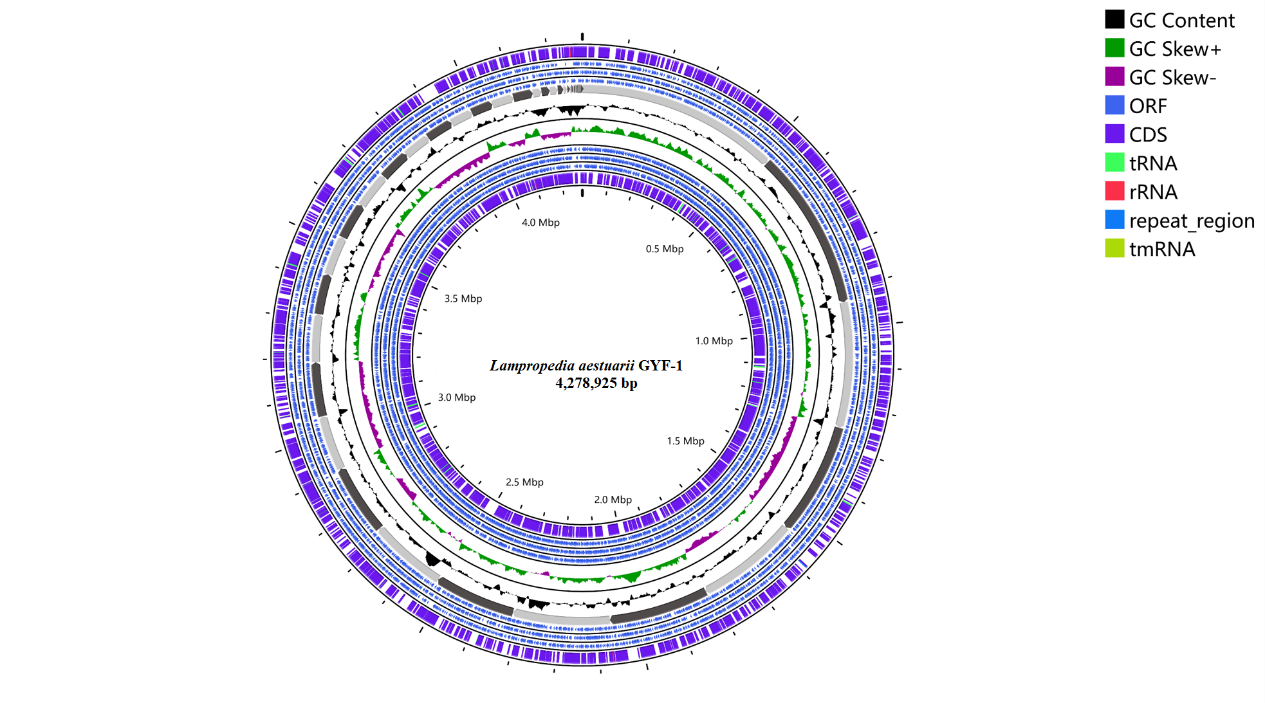


**Figure S1.** Draft genome of *L. aestuarii* GYF-1

A graphical circular map of the genome performed with Proksee. From outside to center, ring 1 and ring 11 show coding sequences, tRNA, rRNA, repeat region, and tmRNA on both the forward and reverse strand. Ring 2-4 and ring 8-10 in blue show ORFs, depending on strand orientation. The ring 5 in dark grey and light grey shows all the scaffolds. Ring 6 in black shows GC content, and ring 7 in green or rose red shows GC skew.


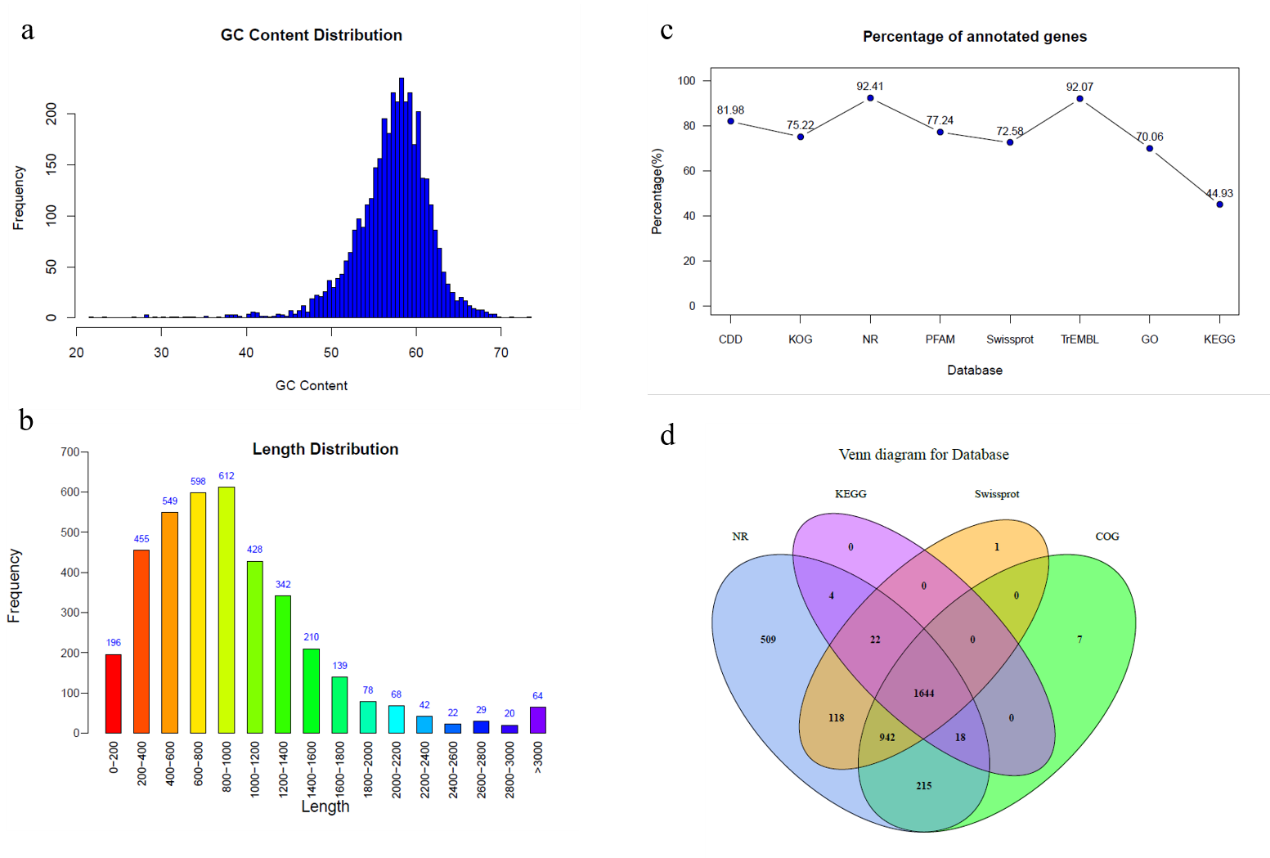


**Figure S2.** Genome features of *L. aestuarii* GYF-1

1. GC content distribution; B) Percentage of annotated genes in indicated databases; C) distribution of gene length predicted in strain GYF-1 genome; and D) Venn diagram indicated the number of genes annotated in each database.


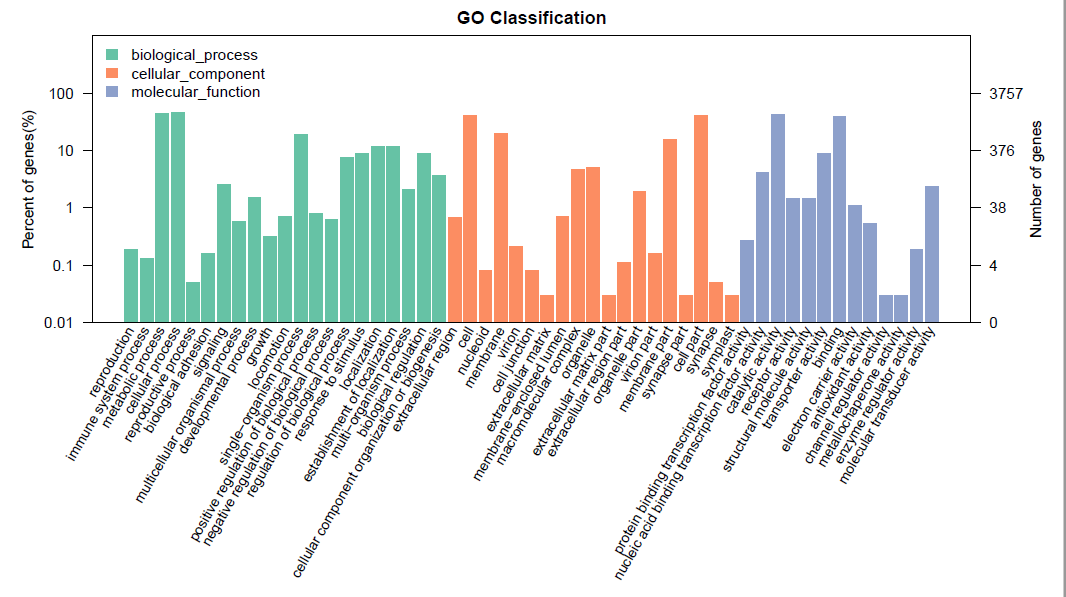


**Figure S3.** GO classification

GO annotation of predicted genes in *L. aestuarii* GYF-1.


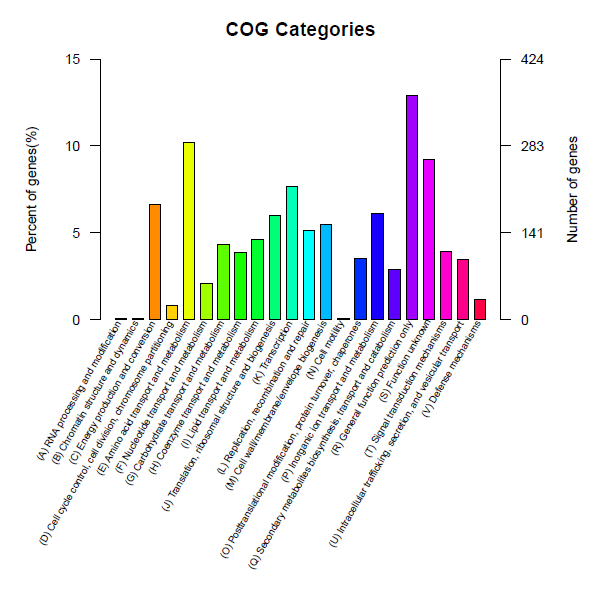


**Figure S4.** COG categories

COG categories of predicted genes in *L. aestuarii* GYF-1.


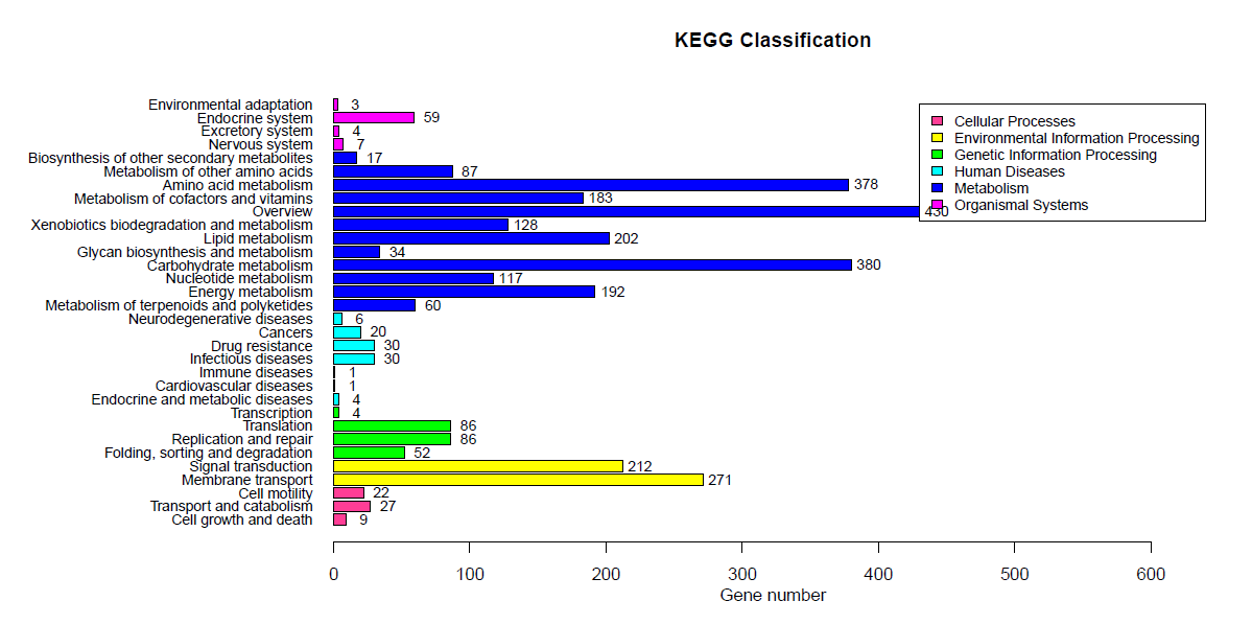


**Figure S5.** KEGG classification

KEGG classification of predicted genes in *L. aestuarii* GYF-1.


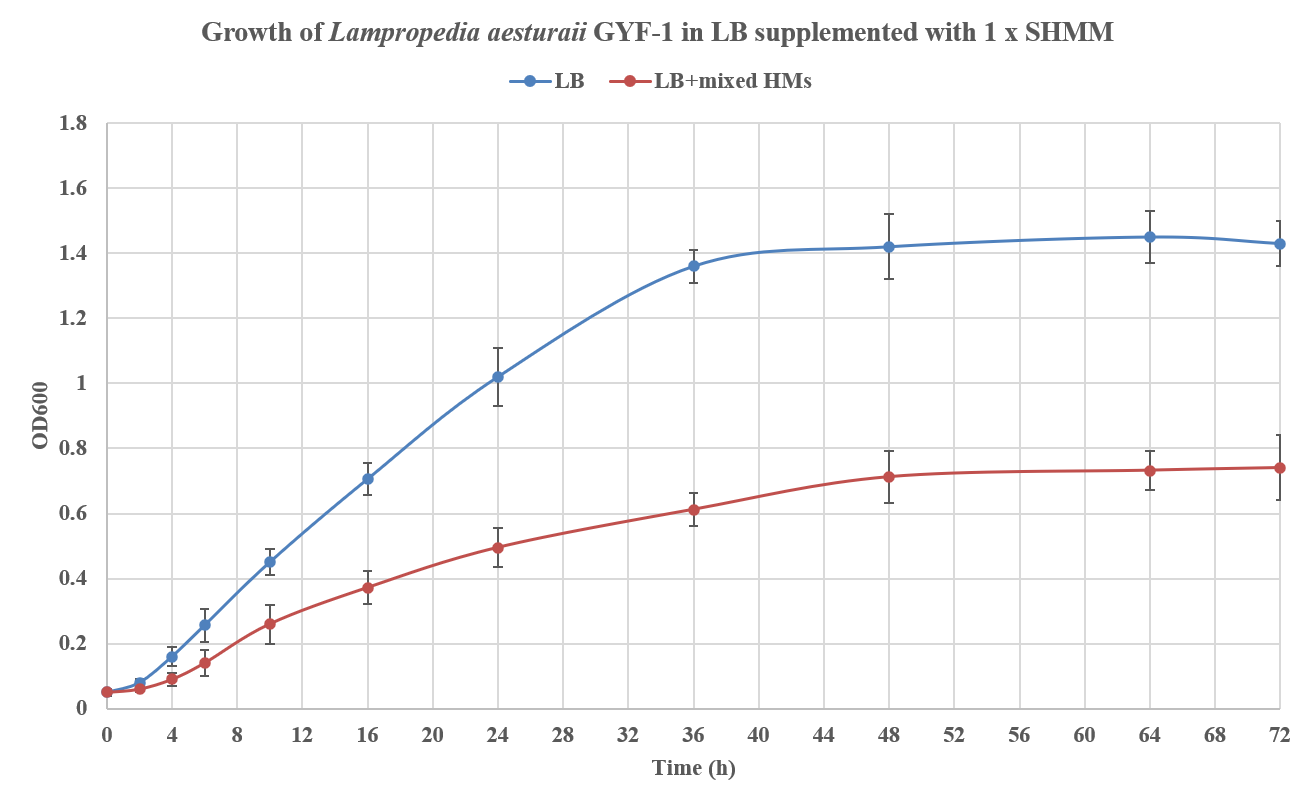


**Figure S6.** Growth of strain GYF-1 in mixed heavy metal medium

The 100 ml of LB or LB supplemented with 1 × SHMM was inoculated with GYF-1 seeds at final OD_600_ of 0.05 and shaking at 30^o^C for 72 h. The 5 ml of cultures were sampled for measurement of cell growth at indicated time (n=3).


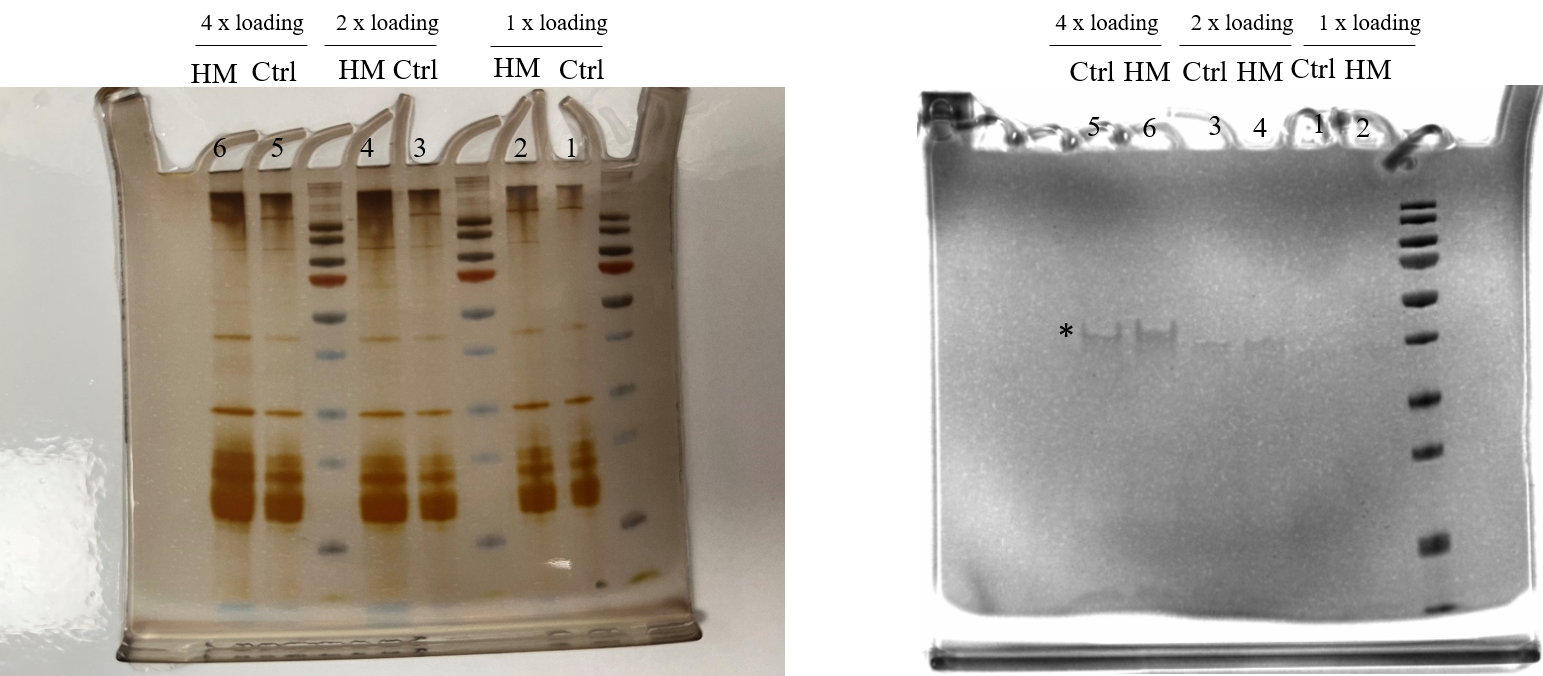


**Figure S7.** Silver staining and Coomassie blue staining of lipopolysaccharides

Silver staining (left) of extracted LPS, and coomassie blue R-250 staining (right) used to examine protein contamination. 10 mg of cell pellets were subjected to LPS extraction with two biological replicates, 10 μL of each sample (1 × loading) loaded to each lane for relative LPS level comparison, * indicated non-specific bands.


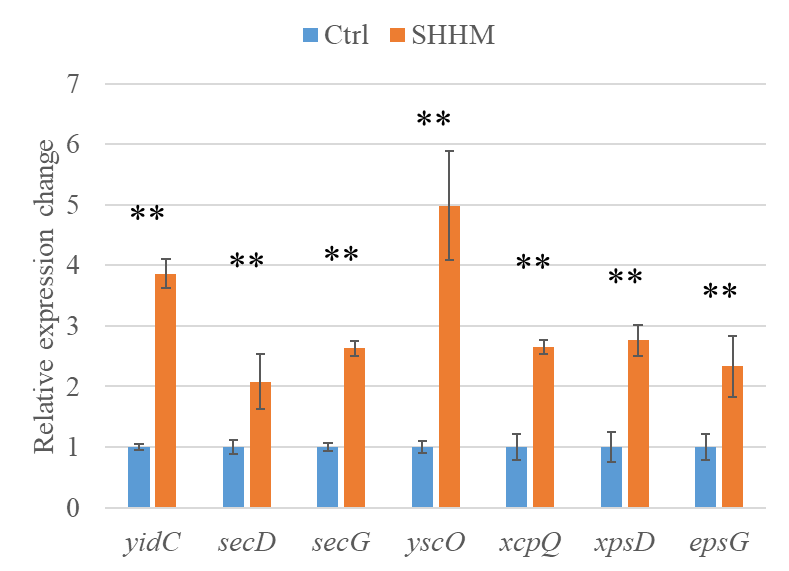


**Figure S8.** Relative gene expressions response to mixed heavy metals

Relative gene expressions involved in secretory pathway. Bars represented the mean and the error bars the standard error of the mean (mean ± SEM)


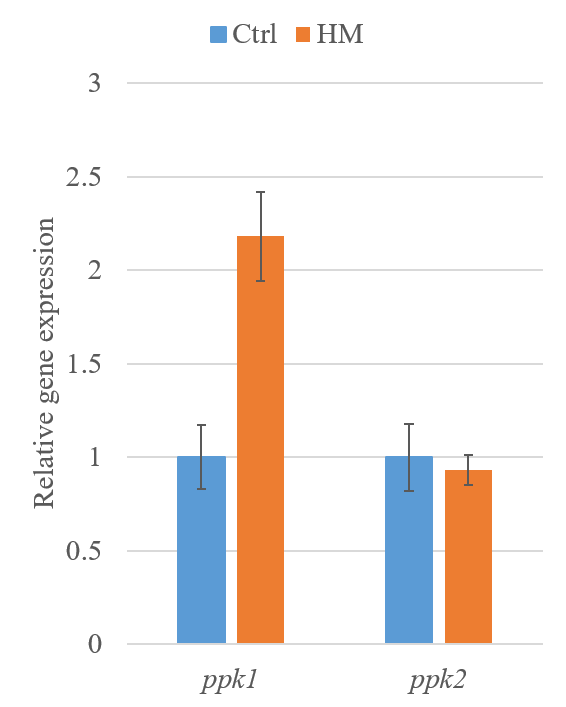


**Figure S9.** Relative expression of polyphosphate kinase.

Relative gene expressions involved in inorganic polyphosphate synthesis. Bars represented the mean and the error bars the standard error of the mean (mean ± SEM).
